# Identification of Bacteria in Two Food Waste Black Soldier Fly Larvae Rearing Residues

**DOI:** 10.3389/fmicb.2020.582867

**Published:** 2020-11-23

**Authors:** Moritz Gold, Fabienne von Allmen, Christian Zurbrügg, Jibin Zhang, Alexander Mathys

**Affiliations:** ^1^Sustainable Food Processing Laboratory, Department of Health Science and Technology, Institute of Food, Nutrition and Health, ETH Zürich, Zurich, Switzerland; ^2^Department Sanitation, Water and Solid Waste for Development (Sandec), Eawag: Swiss Federal Institute of Aquatic Science and Technology, Dübendorf, Switzerland; ^3^State Key Laboratory of Agricultural Microbiology, National Engineering Research Center of Microbial Pesticides, College of Life Science and Technology, Huazhong Agricultural University, Wuhan, China

**Keywords:** *Hermetia illucens*, microbiota, insects, bioconversion, waste, frass, probiotics

## Abstract

Significant economic, environmental, and social impacts are associated with the avoidable disposal of foods worldwide. Mass-rearing of black soldier fly (*Hermetia illucens*) larvae using organic wastes and food- and agro-industry side products is promising for recycling resources within the food system. One current challenge of this approach is ensuring a reliable and high conversion performance of larvae with inherently variable substrates. Research has been devoted to increasing rearing performance by optimizing substrate nutrient contents and ratios, while the potential of the substrate and larval gut microbiota to increase rearing performance remains untapped. Since previous research has focused on gut microbiota, here, we describe bacterial dynamics in the residue (i.e., the mixture of frass and substrate) of black soldier fly larvae reared on two food wastes (i.e., canteen and household waste). To identify members of the substrate and residue microbiota, potentially associated with rearing performance, bacterial dynamics were also studied in the canteen waste without larvae, and after inactivation by irradiation of the initial microbiota in canteen waste. The food waste substrates had similar microbiota; both were dominated by common lactic acid bacteria. Inactivation of the canteen waste microbiota, which was dominated by *Leuconostoc*, *Bacillus*, and *Staphylococcus*, decreased the levels of all rearing performance indicators by 31–46% relative to canteen waste with the native microbiota. In both food waste substrates, larval rearing decreased the bacterial richness and changed the physicochemical residue properties and composition over the rearing period of 12 days, and typical members of the larval intestinal microbiota (i.e., *Providencia*, *Dysgonomonas*, *Morganella*, and *Proteus)* became more abundant, suggesting their transfer into the residue through excretions. Future studies should isolate members of these taxa and elucidate their true potential to influence black soldier fly mass-rearing performance.

## Introduction

Significant economic, environmental, and social impacts are associated with the avoidable disposal of foods worldwide (Gustavsson et al., 2011; Papargyropoulou et al., 2014; Chen et al., 2020). Measures to reduce food loss and waste amount exceeding 1.3 billion tons per year (Gustavsson et al., 2011) include avoiding surplus food production, followed by redistribution and reuse of surplus foods (Papargyropoulou et al., 2014). Once produced, food loss and wastes should be recycled by using them as animal feed (Dou et al., 2018) or compost (Li et al., 2013), and extraction of energy should be the least preferred approach (Papargyropoulou et al., 2014). In recent years, mass rearing of the black soldier fly (*Hermetia illucens* L., Diptera: Stratiomyidae) larvae (BSFL) has emerged as an additional solution for food waste recycling (Gold et al., 2018; Zurbrügg et al., 2018; Berggren et al., 2019; Varelas, 2019). BSFL convert a range of organic wastes (e.g., food waste, animal manure) and food- and agro-industry side products (e.g., breweries, food processing industry) (Nyakeri et al., 2017; Barragán-Fonseca et al., 2018; Lalander et al., 2019; Gold et al., 2020a) into larval biomass and a compost-like residue (i.e., mixture of frass and substrate). The larval biomass is rich in proteins and lipids, and thus, serves as a raw material for various applications within the food system, such as proteins and lipids in feeds for pets (Bosch et al., 2014) and livestock (e.g., fish, poultry, swine) (Barragán-Fonseca et al., 2017; Wang and Shelomi, 2017), and processing of the larval exoskeleton into chitosan (Hahn et al., 2019). Next to this recycling of waste nutrients, according to circular economy principles (Cappellozza et al., 2019), waste treatment by BSFL (Ermolaev et al., 2019; Mertenat et al., 2019; Pang et al., 2020) and animal feed products (Smetana et al., 2016, 2019) with BSFL can have lower environmental impact than the *status quo* (i.e., composting and commercial feed ingredients such as fish meal).

One current challenge for BSFL rearing is to obtain reliable and high rearing performance (e.g., >200 mg for harvested BSFL) (Gold et al., 2018). Researchers have previously improved rearing performance by optimizing substrate nutrient contents and ratios (Nyakeri et al., 2017; Barragán-Fonseca et al., 2018; Gold et al., 2020a), however, few studies exist regarding the manifold roles in which BSFL-associated microbiota may influence rearing performance (De Smet et al., 2018). BSFL guts, rearing substrates and residues (i.e., the mixture of frass and substrate) all have rich and diverse microbiomes (Bruno et al., 2019; Klammsteiner et al., 2020) varying due to different biotic (e.g., initial rearing substrate microbiome) and abiotic (e.g., temperature) factors among rearing systems (Wynants et al., 2019; Raimondi et al., 2020). Similar to many insects (Douglas, 2009; Engel and Moran, 2013; Lee and Brey, 2013), Dipteran larvae such as those of *Drosophila melanogaster* (Diptera: Drosophilidae) and *Musca domestica* (Diptera: Muscidae) engage in complex interactions with their gut microbiota, as these influence larval immunity (Broderick and Lemaitre, 2012) and metabolism (Shin et al., 2016), growth signaling (Storelli et al., 2011), and nutrient provision (Zurek and Nayduch, 2016). Microbiota associated with BSFL may have similar functions (Ao et al., 2020) considering their similar ecological niche and phylogenetic order (Gold et al., 2018; Zhan et al., 2020). Identification and manipulation (e.g., by addition of bacterial mixtures) of microbiota in BSFL rearing is an additional promising approach to increase rearing performance, next to the optimization of substrate nutrient contents and ratios.

Members of the BSFL gut microbiota (e.g., *Bacillus natto*, *Bacillus subtilis*, *Lactobacillus buchneri*, and *Kocuria marina*) can increase performance when added to rearing substrates (Yu et al., 2011; Xiao et al., 2018; Rehman et al., 2019; Somroo et al., 2019; Mazza et al., 2020). Members of the substrate and residue microbiota are an additional yet unexplored pool of potentially beneficial bacteria. Fly larvae may support and sustain certain microbiota in the substrate and residue to favor decomposition and digestibility (Zhao et al., 2017) and thus larval development (Storelli et al., 2018) and also protect from other insects and microbes competing for the same resources (Bernard et al., 2020). Bacterial candidates associated with rearing performance could be identified by studying microbiota throughout the rearing time in the substrate and rearing residue. For BSFL, previous studies have typically only determined the bacterial community in the initial substrate as well as the final residue. Jiang et al. (2019) were the first to determine bacterial community dynamics throughout one rearing cycle and found that BSFL rearing affects and alters the substrate bacterial community over time to increase the capacity to decompose the substrate. Lalander et al. (2013) and Erickson et al. (2004) also reported a reduction in certain bacteria during BSFL rearing, and Cai et al. (2018) and Liu et al. (2020) reported a reduction in antibiotic resistance genes in bacteria. These results contradict the findings of Bruno et al. (2019), who did not identify a significant influence of BSFL on substrate microbiota during rearing. Changing the bacterial community in the rearing substrate may also be beneficial for rearing of insects for food and feed applications, when considering that the substrate microbial community may include human and animal pathogens (e.g., *Bacillus cereus* and *Enterococcus faecalis*) (Lalander et al., 2013; Van der Fels-Klerx et al., 2018; Wynants et al., 2019).

The aim of this study was to identify groups of bacteria potentially associated with BSFL residues and the rearing performance. We assessed the bacterial community dynamics when using two food waste substrates during BSFL rearing. We also studied the bacterial community dynamics in food waste after inactivation by irradiation of the initial microbiota in food waste with and without BSFL. We hypothesized that an inactivation of the initial microbial community in the substrate should decrease rearing performance, revealing that some important bacteria are associated with rearing performance. In addition, we hypothesized that certain groups of bacteria become more abundant during BSFL rearing in comparison to controls without larvae.

## Materials and Methods

### Food Waste Substrates

Two types of food waste were collected in containers treated with 70% ethanol. Canteen waste included a mixture of discarded pasta, meat, fish, bread, and vegetables from the Polyterrasse canteen at ETH Zürich in Switzerland. Household waste included discarded fruit peels, vegetables, eggs, bread, herbs, and food leftovers collected from a household organic waste bin in Zürich. Each collected substrate mixture was homogenized with a kitchen blender to mimic typical waste processing before BSFL rearing (Dortmans et al., 2017). Pictures of the fresh and homogenized rearing substrates are included in Supplementary Table 1. Following homogenization, the rearing substrates were stored at 4°C for 48 h. During this storage time, part of the canteen waste was sterilized with a high-energy electron beam. This substrate was fed to BSFL in parallel to the non-sterile food wastes to assess the influence of the loss of the initial substrate microbiota on rearing performance and bacterial dynamics. Irradiation was completed by a commercial provider (Leoni Studer AG, Däniken, Switzerland) with a 10 MeV electron beam (Rhodotron TT300, IBA Corp., Louvain-la-Neuve, Belgium) at a dose of 32 kGy in accordance with the ISO 11137-3:2017 standard [International Organization for Standardization (ISO), 2017]. These treatment conditions produced sterile substrates without microbial growth (Gold et al., 2020b).

Since substrate composition influences microbial communities and BSFL rearing performance, substrate gross nutrient composition, pH, and moisture content were determined using standard procedures described in detail in Gold et al. (2020a). The pH was measured with a portable meter and the pH probe HQ40d (Hach Lange GmbH, Rheineck, Switzerland). Moisture and organic matter were determined using an automatic thermogravimetric instrument (TGA-701, Leco, St. Joseph, MI, United States). Nitrogen content was determined using a C/N analyzer (Type TruMac CN, Leco). Glucose (D-Glucose GOPOD K-GLUC, Megazyme, Wicklow, Ireland) and starch (Total Starch Assay K-TSTA, Megazyme), and fructose (Available Carbohydrates K-ACHDF, Megazyme) were determined using commercial enzyme assays. For fructose determination, absorbance differences of >0.07 were used instead of >0.1, as recommended by the manufacturer. Neutral and acid detergent fibers were assessed using a fiber bag system (Fibretherm, Gerhardt Analytical Systems, Königswinter, Germany). Lipids were analyzed by Eurofins Scientific, Schönenwerd, Switzerland, according to Regulation (EC) No 152/2009 (European Commission, 2009). Protein was estimated by multiplying the nitrogen results with 5.4 (based on results for meat, fish, cereals, and vegetables) (Mariotti et al., 2008), and the caloric content was estimated by multiplying the mean lipid, non-fiber carbohydrate, and protein results with their gross caloric content of 9.4, 5.4, and 4.1 kcal/g, respectively (Merrill, 1973; Wu, 2016). Hemicellulose content was determined as the difference between the neutral and acid detergent fibers. The sum of glucose, fructose, and starch was assumed to reflect the total non-fiber carbohydrate content.

### Fly Larva Rearing

BSFL were reared on homogenized food waste substrates using the following experimental setup. BSFL were obtained from a colony operated at Eawag (Dübendorf, Switzerland) since 2017, based on the protocol of Dortmans et al. (2017). The hatched larvae were fed *ad libitum* with poultry feed (UFA 625, UFA AG, Herzogenbuchsee, Switzerland) for 7–9 days to a weight of 0.5 mg dry mass (DM)/larva. Thereafter, larvae were manually separated from the poultry feed residue and 12 replicates with approximately 200 larvae per replicate were prepared for each treatment. To eliminate possible contaminations by airborne microbes, cross-contamination between substrates, and contamination with human microbes during rearing, larvae were reared in an one-time-feeding bench-scale batch experiment. Larvae were placed in sterile plastic containers (diameter: 100 mm, height: 80 mm) (O118/80, Eco2 NV, Ophasselt, Belgium) with substrates at a feeding rate of 22 mg DM/larva per day for 12 days, resulting in a larval density of 2.5 larvae/cm^2^. Plastic containers were covered with lids (OD118 Filter XL, Eco2 NV, Ophasselt) permitting air flow, while being impermeable to microbes. Containers were placed in a random order in a climate chamber (HPP 260, Memmert GmbH, Büchenbach, Germany), providing a microclimate of 28°C and 70% relative humidity. Temperature was automatically recorded every 10 min in the substrate/residue of one replicate per treatment (DS1922L iButton, Maxim Integrated, San Jose, CA, United States).

For each treatment, every 3 days, for a total of 12 days, three containers were removed from the climate chamber. One residue sample was collected per removed container to determine the physicochemical (see section “Physicochemical Properties and Composition of the Residue”) and microbial parameters in the residue (see section “Microbial Counts and Bacterial Communities”). Larvae were manually separated from the residue, cleaned with tap water, and dried with paper towels. Larvae were counted, weighed, freeze-dried and then stored at 4°C before larval protein content (see section “Microbial Counts and Bacterial Communities”) measurement. Residue samples were analyzed for water activity and pH, thereafter freeze-dried and stored to later measure other physicochemical parameters. Weight loss in larvae and residue samples during freeze drying was used to correct all results for moisture content. All manipulations with rearing containers and collection of substrate/residue samples for microbial parameters were performed using sterile techniques under a laminar flow cabinet.

### Controls With No Larvae

Sterile and non-sterile canteen waste without larvae underwent experimental and environmental conditions identical to those used for BSFL rearing. After 12 days in sterile containers in the climate chamber, samples were collected and processed in the same way as the rearing residue samples.

### Physicochemical Properties and Composition of the Residue

Changes in the residue composition were measured through physicochemical parameters which are relevant for microbial growth. Portable meters were used to measure water activity (HygroPalm23-AW, Rotronic, Bassersdorf, Switzerland) and pH (HQ40d, Hach Lange GmbH) in the fresh residue samples. A thermogravimetric instrument (TGA-701, Leco) and a C/N analyzer (Type TruMac CN, Leco) were used to measure moisture and organic matter, and carbon and nitrogen on the freeze-dried residue samples, respectively.

### Rearing Performance

Residue and larval dry weights as well as larval protein content were used to calculate typical rearing performance indicators per replicate. Larval weight, bioconversion rate, and waste reduction were calculated according to Gold et al. (2020a). The total larval protein per biological replicate (which had 200 larvae) was calculated using equation 1.

(1) $$\begin{array}{c} \begin{array}{c} \text{Total larval}\\ \text{protein} \end{array}\left(\frac{\text{g DM protein}}{\text{replicate}}\right)=\text{larval weight}\left(\frac{\text{g DM}}{\text{larva}}\right)\text{x}\\ \text{larval protein content }\left(\frac{\text{g DM protein}}{100\text{ g DM}}\right)\text{x}\frac{200\text{ larvae}}{\text{replicate}} \end{array}$$

Each larval protein content was estimated as nitrogen content × 4.67, as proposed by Janssen et al. (2017). Nitrogen was measured on freeze-dried samples using a C/N analyzer (Type TruMac CN, Leco) and corrected for residual moisture with thermogravimetric determinator (TGA-701, Leco).

### Microbial Counts and Bacterial Communities

Culturable microbial counts (i.e., CFU: colony forming units) in the substrate and residue were estimated using plate counts from a dilution series. Microbes were extracted from samples (10 g) by 2 min Stomacher treatment in a medium for recovery of organisms (Difco Maximum Recovery Diluent, BD Diagnostics, Le Pont-de-Claix, France). For each sample, 50 μL of the dilution series were spread in duplicate on Petri dishes (diameter: 90 mm) divided into four quadrants. Since we partially recorded colonies within the representative range of 20–250 for different dilutions and replicate plates, counts in the Stomacher-homogenate were calculated using equation 2 (Maturin and Peeler, 2001)

(2) $$\frac{C\,F\,U}{m\,L}=\frac{\sum \text{c}}{\text{V}\times \left(1\,{\text{n}}_{1}+0.1\,{\text{n}}_{2}\right)\times \text{d}}\;$$

where Σ*c* is the number of colonies on all plates, *V* is the volume added to each plate (0.05 mL), n_i_ is the number of quadrants counted of the *i*_th_ dilution, and *d* is the dilution. Total viable counts (TVC) were determined using standard agar (15 g/L Agar, VWR International, Leuven, Belgium; 30 g/L Tryptic Soy Broth No. 2, Sigma Aldrich GmbH, Buchs, Switzerland), lactic acid bacteria (LAB) on De Man, Rogosa, and Sharpe Agar (MRS, VWR International), and fungi on Dichloran Rose Bengal Chloramphenicol Agar (DRBC, Sigma Aldrich GmbH) after incubation at 30°C for 20 to 48 h. Media and incubation conditions were selected based on the manufacturer’s directions and previous work by Wynants et al. (2019).

**FIGURE 1:**
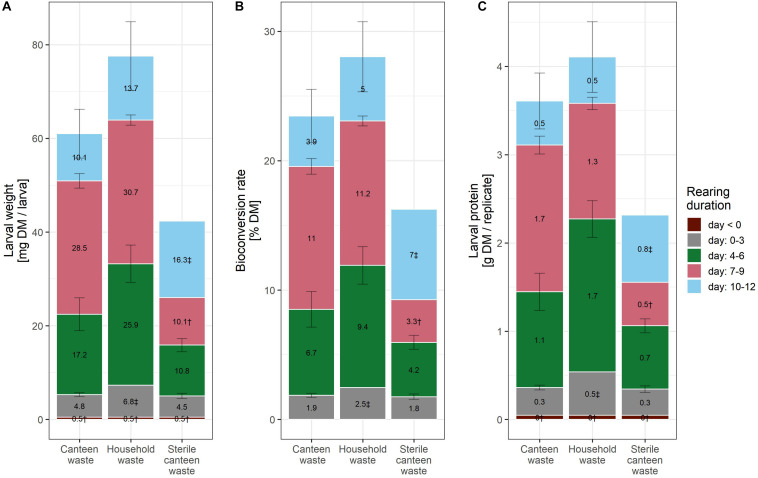
Rearing performance of BSFL on canteen and household food waste. Bar plot of larval weight **(A)**, bioconversion rate **(B)**, and larval protein per replicate **(C)**. The bar plot label shows the mean increase in the levels of the performance indicator between the measurement days. ^†^ (*n* = 1) and ^‡^ (*n* = 2) indicate results with fewer than three biological replicates.

**FIGURE 2:**
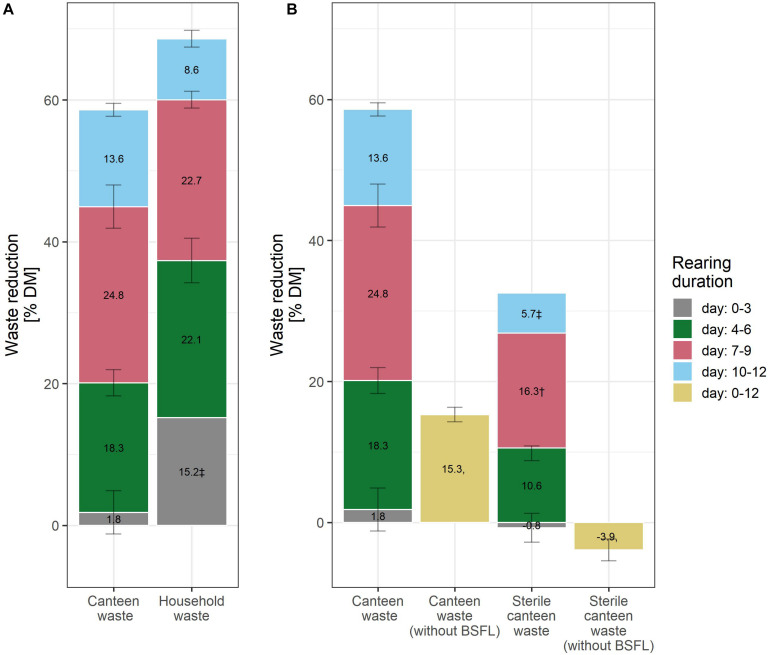
Waste reduction during BSFL rearing with canteen and household food waste **(A)**, and on canteen waste without larvae, and sterile canteen waste with and without larvae **(B)**. The bar plot label shows the mean increase in the levels of the performance indicators between the measurement days. ^†^ (*n* = 1) and ^‡^ (*n* = 2) indicate results with fewer than three biological replicates.

The bacterial community was determined by high-throughput 16S rRNA gene sequencing using the MiSeq Illumina platform. Total genomic DNA was extracted from 0.2 g of substrate (in single) or residue (single to quadruplicate per substrate and sampling day, see Figures 1, 2) sample using the DNeasy PowerSoil Kit (QIAGEN, Hilden, Germany) with one modification. To enhance DNA extraction, three sterile metal beads (diameter: 3 mm; Uiker AG, Freienbach, Switzerland) were added to each PowerBead Tube with the lysis buffer and homogenized with a Bead Ruptor (Omni International, Kennesaw GA, United States; speed 5.5, 2 × 20 s with 30 s break between rounds). Purity (NanoDrop ND 1000 Spectrophotometer, Thermo Scientific, Wilmington MA, United States) and concentration Qubit dsDNA HR Assay Kit on a Spark 10 M microplate reader (Tecan, Männedorf, Switzerland) of the extracted DNA was determined. Library preparation followed a two-step protocol. Limited-cycle PCR was conducted in a 25 μL reaction volume using KAPA HiFi HotStart ReadyMix (12.5 μL) (Kapa Biosystems, Wilmington, MA, United States), template DNA (5 μL), forward and reverse primer (0.75 μL; 0.3 mM each), and molecular-grade water (6 μL). All primers included a hexanucleotide barcode and Illumina adapters (Illumina Inc., San Diego, CA, United States). The prokaryotic V3-V4 hypervariable region was amplified in triplicate using the primer pair 341F (5′- CCT ACG GGN GGC WGC AG 3′) and 806R (5′- GGA CTA CNV GGG TWT CTA AT - 3′). PCR conditions were an initial denaturation at 95°C for 300 s, 1 cycle at 98°C for 60 s, 26 cycles of 98°C for 20 s, 51°C for 20 s, and 72°C for 12 s, and a final extension at 72°C for 120 s (Hugerth et al., 2014). Negative controls were run by replacing template DNA with molecular grade water. A positive control was run by replacing template DNA with a known mixture of bacterial DNA (i.e., mock sample). Products from the first PCR were pooled, cleaned using solid-phase reversible immobilization beads (ETH Zürich Genetic Diversity Center, Zurich, Switzerland) and used as a template for the second PCR to attach dual indices using the Nextera XT Index Kit v2 (Illumina Inc.). Index PCR included the first PCR product (2 μL), KAPA HiFi HotStart ReadyMix (10 μL), molecular grade water (4 μL), and Nextera indexing primers (2 μL). PCR conditions were an initial denaturation at 95°C for 180 s, 10 cycles at 95°C for 30 s, 55°C for 30 s, and 72°C for 30 s, and a final extension at 72°C for 300 s. Index PCR products were cleaned and DNA concentration was determined using the High Sensitivity D1000 Kit on a 2200 TapeStation (Agilent Technologies Inc., Santa Clara, CA, United States). Cleaned PCR products were pooled equimolar to a library concentration of 2 nM. The concentration and purity of the pooled library were controlled using a Qubit Fluorometer (Invitrogen Q32857, Carlsbad, CA, United States) and the TapeStation, respectively. Paired-end sequencing was performed using 19 pM of the prepared library in a single MiSeq 2 × 300 bp flow cell, using the MiSeq Reagent Kit v3 and a 10% PhiX concentration according to the manufacturer’s directions (Illumina Inc.).

### Bioinformatics

Initial quality control of the sequencing data was conducted using FastQC (version 0.11.2). Subsequent bioinformatics data preparation included trimming of read ends with USEARCH (version 11.0.667) and merging of pairs into amplicons with FLASH (Magoč and Salzberg, 2011). Following removal of primer sites with USEARCH, quality-filtering was performed with the PRINSEQ-lite (version 0.20.4) (Schmieder and Edwards, 2011). The resulting high-quality reads were de-noised and clustered into zero-radius operational taxonomic units (ZOTUs) using the UNOISE3 algorithm (Edgar, 2016b). The taxonomic origin of each ZOTU was determined with the SINTAX algorithm (version 11.0.667) (Edgar, 2016a) in USEARCH using Silva 16S (V128) as the reference database. Taxonomic assignments were considered reliable when bootstrap confidence values exceeded 0.85.

### Downstream Data Analyses

Data were analyzed using R version 312 3.6.2 (R Core Team, 2020). Rare ZOTUs with less than 10 total counts and samples with less than 2000 reads (i.e., the highest number of reads in the control samples) were removed before downstream analyses. ZOTUs belonging to the phylum *Cyanobacteria* and to the family *Mitochondria* were also removed, as they likely belonged to eukaryotic 16S rRNA (Michelou et al., 2013), given the plant-based nature of the rearing substrates. We abstained from statistical analyses among sampling days for all parameters due to the small number of replicates (*n* = 1–3). Instead, we compared the results visually or using the mean and standard deviation (when *n* >2, difference between values for *n* = 2). The mean and standard deviation were calculated for rearing performance indicators, physicochemical residue parameters, and microbial counts. Differences in rearing performance indicators between measurement days were calculated by subtracting the mean values. Pearson correlation coefficients (*p* < 0.01) were calculated following visual assessment of normality (see Supplementary Figures 2, 3) to identify linear dependencies between rearing performance indicators and the physicochemical residue composition.

**FIGURE 3:**
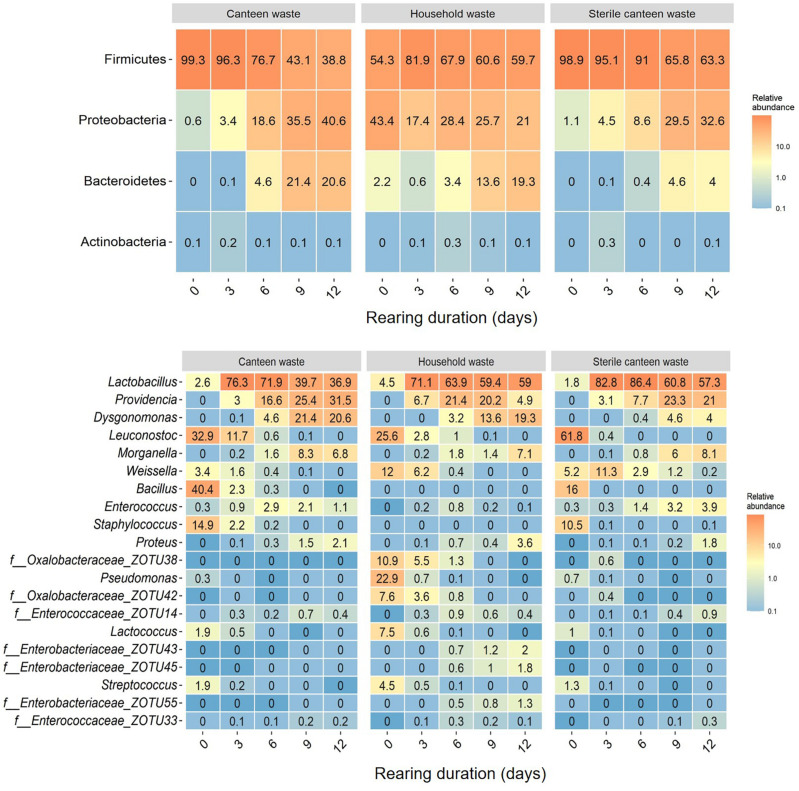
Substrate and residue bacterial community at different days (0–12 days) of BSFL rearing. Heatmaps of the top phyla **(top)** and top 20 genera **(bottom)** based on the relative abundance of ZOTUs in all samples. Relative abundances are the mean of replicate samples rounded to one digit. If no clear assignment to a genus was possible, the family assignment is shown together with the ZOTU.

Heat maps at the phylum and genus levels were produced in *ampvis2* (Andersen et al., 2018) after conversion of reads into percent abundance per sample. The same package was used to identify shared ZOTUs between samples with Venn diagrams (frequency cutoff >80% and abundance cutoff >0.01%). Alpha diversity (i.e., observed richness, Chao1, Shannon index, and Simpson Index) and beta diversity [i.e., non-metric multidimensional scaling (NMDS)] were calculated in *phyloseq* (McMurdie and Holmes, 2013). NMDS was used to illustrate the bacterial dynamics of ZOTUs that accounted for more than 1% of relative abundance in all samples using weighted UniFrac distance to account for phylogenic distances between ZOTUs. Distance-based redundancy analysis (dbRDA) was performed on the same data in *vegan* (Oksanen et al., 2019) with the capscale function using the Bray-Curtis Dissimilarity matrix to determine correlations between physicochemical residue properties and composition, rearing performance, and bacterial community dynamics. Prior to analysis, parameters with co-linearity were excluded from the data with a Pearson correlation matrix (see Supplementary Figure 1) and variance inflation factors (VIF) using the *usdm* function. A VIF value > 5 indicated multi-collinearity. The remaining parameters were scaled and centered. The significance of the model and the correlation of each parameter with the bacterial community was determined by the permutation test (1000 iterations) with a *p*-value < 0.05, denoting significance.

## Results

### Waste Nutrient Composition

The household and canteen waste rearing substrates had similar protein contents, but canteen waste was richer in hemicellulose, lipids, and non-fiber carbohydrates (Table 1). Household waste had more cellulose and lignin, as well as glucose and fructose, that was almost absent in the canteen waste.

**TABLE 1:** Mean nutrient composition of canteen and household waste used for rearing.

		*Non-fiber carbohydrates*				*Fiber*					
	Protein	Total	Glucose	Fructose	Starch	Total	Hemicellulose	Cellulose and lignin	Lipids	P:NFC^1^ ratio	Caloric^2^ content
Canteen waste	15.3 (0.4)	38.6 (0.1)^‡^	0.3 (0.1)	0 (0.0)^‡^	37.2 (2.0)	36.1 (2.3)^‡^	26.9 (1.2)^‡^	9.1 (1.1)^‡^	24.5^†^	1:2	471
Household waste	16.3 (1.1)	29.5 (4.1)^‡^	5.9 (0.7)	7.5 (1.5)^‡^	16.5 (2.4)	22.9 (1.5)^‡^	8.0 (1.1)^‡^	14.9 (0.4)^‡^	18.1^†^	1:2	412

*Nutrients are in percent dry mass and caloric content in kcal per 100 g dry waste. In parenthesis: standard deviation for samples where *n* ≥ 3 and differences between analyses where *n* = 2. ^1^ P:NFC = ratio of protein to non-fiber carbohydrates (NFC). ^2^ gross caloric content of protein, NFC, and lipids. ^†^*n* = 1, ^‡^*n* = 2.*

### Rearing Performance

Considering the mean and standard deviation, the bioconversion rate and larval protein were similar between the two rearing substrates (Figures 1B,C), but larval weight (Figure 1A) and waste reduction (Figure 2A) were higher for household than for canteen waste. Following 12 days of rearing, mean (standard deviation) bioconversion rate and larval protein were 28.0 (2.7)% DM and 4.1 (0.4) g protein/replicate for household waste, and 23.5 (2.1)% DM and 3.6 (0.3) g protein/replicate for canteen waste. Larval weight and waste reduction were higher for household than for canteen waste: 77.6 (7.3) mg DM and 68.6 (1.2)% DM for household waste, and 61.0 (5.2) mg DM and 58.6 (1.0)% DM for canteen waste.

Inactivation of the initial canteen waste microbiota by irradiation reduced rearing performance (Figures 1, 2). In rearing with sterile canteen waste, larval weight, bioconversion rate, larval protein levels, and waste reduction were reduced by 18.7 mg DM, 7.3% DM, 1.3 g protein/replicate, and 26.8% DM, respectively. Without larvae, 15.3 (1.0)% DM was lost from canteen waste, and −3.9 (1.6)% DM was lost from sterile canteen waste (Figure 2B).

### Physicochemical Residue Composition

Rearing performance indicators were positively correlated with residue moisture content (*r* = 0.75–0.86, *p* < 0.01) and pH (*r* = 0.76–0.81, *p* < 0.01) (Table 2). Moisture and nitrogen content (Table 2) increased throughout the rearing experiment in the canteen and household waste residue in comparison to the substrates. The residue pH decreased from the substrate value in the first half of rearing, and then increased above the initial substrate value in the second half of rearing. Residue organic matter had different trends for the two rearing substrates. It decreased from the value in the household waste, but not in the canteen waste substrate. Values for carbon (range: 49.2–56.0% DM), water activity (range: 0.95–0.99), and temperature (range: 27.5–29.9°C) showed low variability throughout the rearing experiment from the initial value (≤± 5%, see Supplementary Table 2). The residue composition changed much less when the initial canteen waste microbiota was inactivated. Except for the residue moisture content and the carbon to nitrogen ratio, the residue composition was ≤± 5% relative to the initial value in the substrate.

**TABLE 2:** Physicochemical properties, composition, microbial counts, and bacterial community alpha diversity (i.e., richness and diversity) in the substrates and residues.

Substrate	Day	Moisture content	pH	Nitrogen	C/N	Organic matter	TVC	LAB	Fungi	Observed richness	Chao 1	Shannon Index	Simpson’s Index

		%	–	%DM	–	%DM	log_10_/g	log_10_/g	log_10_/g	–	–	–	–
***BSFL rearing***													
Canteen waste	0	69.5 (0.2) ^‡^	4.4 (0.0) ^‡^	2.9^†^	19.3^†^	95.9 (0.0)^‡^	9.2 (0.2)	9.2^†^	5.2 (0.5)	119^†^	140^†^	2.4^†^	0.8^†^
	3	69.0 (1.0)	3.8 (0.0)	3.5^†^	15.2^†^	95.8 (0.1)	8.4 (0.2)	8.4 (0.2)	6.8 (0.0)	113 (14)	148 (45)	2.4 (0.2)	0.8 (0.0)
	6	71.8 (0.3)	3.7 (0.1)	3.4 (0.3)	16.2 (1.4)	95.2 (0.2)	7.7 (0.0)	7.7 (0.1)	6.7 (0.1)	92 (5)	103 (10)	2.3 (0.1)	0.8 (0.0)
	9	77.4 (1.0)	4.6 (0.1)	3.5 (0.1)	16.0 (0.4)	94.1 (0.2)	8.2 (0.0)	8.2 (0.1) ^‡^	7.1 (0.1)	73 (6)	95 (14)	2.5 (0.1)	0.9 (0.0)
	12	81.1 (0.7)	5.6 (0.2)	3.9 (0.1)	14.3 (0.4)	95.0 (0.2)	8.6 (0.5)	8.1 (0.1)	8.7 (0.1)	57 (5)	76 (11)	2.5 (0.1)	0.9 (0.0)
Sterile canteen waste	0	69.5 (0.2)^‡^	4.4 (0.0) ^‡^	2.9^†^	19.3^†^	95.9 (0.0)^‡^	n.a.	n.a	n.a	125^†^	143^†^	2.4^†^	0.8^†^
	3	68.2 (0.5)	3.9 (0.1)	3.1 (0.2)	17.6 (1.4)	95.9 (0.4)	8.6 (0.0)	8.6 (0.1)	6.4 (0.1)	78 (12)	101 (10)	1.5 (0.2)	0.6 (0.1)
	6	69.5 (0.2)	3.8 (0.0)	3.0 (0.1)	18.2 (0.7)	95.4 (0.1)	8.5 (0.1)	8.5 (0.1)	6.9 (0.2)	62 (5)	84 (20)	1.4 (0.2)	0.6 (0.1)
	9	72.0^†^	3.9^†^	3.0^†^	18.0^†^	95.3^†^	8.3^†^	8.0	7.2^†^	53^†^	66^†^	2.0^†^	0.8^†^
	12	73.6 (1.2)^‡^	4.2 (0.0)	3.1 (0.1)	17.9 (0.8)^‡^	95.8 (0.1)^‡^	9.3 (0.1)^‡^	8.2 (0.1)^‡^	9.0 (0.1)^‡^	63 (14)^‡^	89 (30)^‡^	2.4 (0.1)^‡^	0.9 (0.0)^‡^
Household waste	0	76.7 (0.1)^‡^	4.8 (0.0) ^‡^	3.1^†^	16.7^†^	94.3 (0.1)^‡^	9.2 (0.1)	9.0 (0.3)^‡^	5.4 (0.2)	122^†^	147^†^	2.6^†^	0.9^†^
	3	79.4 (0.2)^‡^	3.9 (0.0) ^‡^	3.3^†^	15.8^†^	93.7 (0.0)^‡^	8.6 (0.1)^‡^	8.7 (0.1)^‡^	6.6 (0.1)^‡^	122 (52)^‡^	142 (61)^‡^	2.0 (0.6) ^‡^	0.7 (0.2)^‡^
	6	83.0 (0.5)	3.9 (0.0)	3.2 (0.1)	16.5 (0.4)	92.1 (0.4)	8.0 (0.1)	7.9 (0.1)	6.3 (0.2)	99 (7)	122 (13)	2.3 (0.1)	0.8 (0.0)
	9	88.2 (0.4)	4.5 (0.1)	3.2 (0.1)	15.7 (0.5)	88.8 (0.4)	7.6 (0.1)	7.6 (0.1)	5.0 (0.1)	67 (9)	89 (14)	2.1 (0.0)	0.8 (0.0)
	12	90.2 (0.3)	6.5 (0.6)	3.6 (0.1)	13.8 (0.5)	85.5 (0.6)	10.0 (0.5)	7.5 (0.0)	7.5 (1.3)^‡^	62 (5)	88 (9)	2.2 (0.1)	0.8 (0.0)
***Without BSFL***													
Canteen waste	12	72.2 (0.3)	4.8 (0.3)	3.8 (0.0)^‡^	14.8 (0.2 ^‡^	95.2 (0.1)^‡^	n.a	n.a	n.a	106 (4)^‡^	122 (10)^‡^	2.7 (0.0)^‡^	0.9 (0.0)^‡^
Sterile canteen waste	12	66.9 (0.4)	4.0 (0.1)	3.1 (0.1)	17.2 (0.5)	95.7 (0.0)^‡^	n.a	n.a	n.a	97 (9)	133 (11)	1.6 (0.3)	0.6 (0.1)

*See Supplementary Table 2 for the carbon, water activity, and temperature results. In parenthesis: standard deviation for samples where *n* ≥ 3, differences between analyses where *n* = 2. ^†^*n* = 1, ^‡^*n* = 2, n.a., not analyzed. C/N: carbon to nitrogen ratio. Richness: The number of ZOTUs determined with 16S rRNA gene sequencing based on DNA extracted from samples. Chao 1: the total number of ZOTUs estimated by infinite sampling. A higher number indicates a higher richness (Chao, 1984). Shannon Index: Measure richness and evenness. Increases with community richness and evenness (Shannon and Weaver, 1949; Lemos et al., 2011). Simpson’s Index: Measures community evenness. Index increases as diversity decreases (Simpson, 1949; Lemos et al., 2011).*

### Microbial Dynamics

#### Canteen and Household Waste Rearing Substrates

Canteen and household waste had similar counts of TVC, LAB, and fungi (Table 2). Throughout the rearing experiment, residue microbial counts deviated by ±1–3 log_10_ CFU/g from the counts in the substrate. At the end of the rearing experiment, TVC in the canteen waste residue was 1 log_10_ CFU/g higher and in the household waste residue 1 log_10_ CFU/g lower than that in the substrate. LAB counts decreased by 1–2 log_10_ CFU/g and fungal counts increased by 2–3 log_10_ CFU/g in the residue in comparison to the counts in the substrate.

Considering all 45 samples, gene sequencing using DNA produced a total of 1,783,860 reads, with an average of 44,597 reads/sample, and a total of 275 ZOTUs. Rarefaction curves (see Supplementary Figure 4) demonstrate that samples were sequenced to an extent sufficient to approximate true diversity. The canteen waste and household waste bacterial community consisted of 119 and 125 ZOTUs, respectively (Table 2). Canteen waste was dominated by a few genera of *Firmicutes*, and household waste by *Firmicutes* and *Proteobacteria* (Figure 3). Dominant genera were *Leuconostoc*, *Bacillus*, *Staphylococcus* in the canteen waste and *Leuconostoc*, *Weissella*, *Pseudomonas*, *Oxalobacteraceae*, and *Lactococcus* in the household waste. Sixty two percent of the relative abundance of the two wastes was due to 28 shared ZOTUs, while 49 ZOTUs were unique to canteen waste and 34 ZOTUs were unique to household waste (see Venn diagram in Supplementary Figure 5). Unique high-abundance (>10%) species were from *Pseudomonas* and *Oxalobacteraceae* in household waste and *Staphylococcus* and *Bacillus* in canteen waste.

**FIGURE 4:**
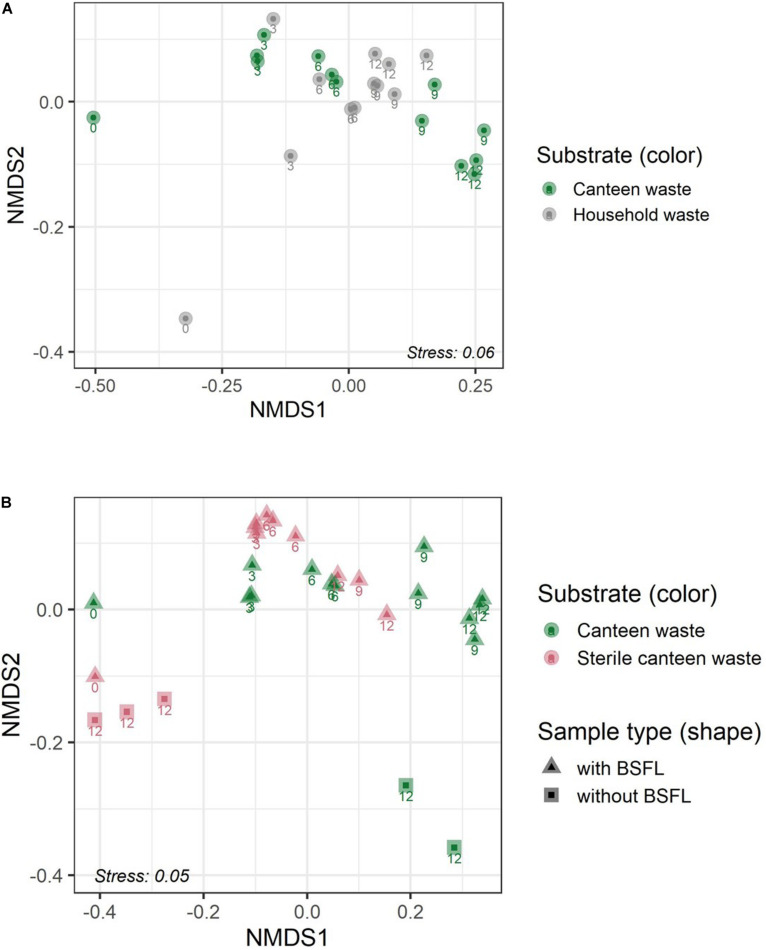
Residue bacterial community dynamics in the two food wastes **(A)** and in canteen waste (sterile, non-sterile) **(B)** during rearing illustrated by non-metric multidimensional scaling (NMDS) of bacterial communities in the substrate and residue based on weighed UniFrac dissimilarity. Numbers adjacent to the symbols indicate the sampling day during BSFL rearing (0–12 days).

The results for the bacterial community alpha and beta diversity demonstrated that the addition of BSFL to the substrates dramatically changed the bacterial richness (Table 2) and community (Figures 3, 4). Community richness decreased on both wastes throughout rearing, and the bacterial community between the two substrates became more similar. Replicate samples clustered well based on UniFrac distances according to rearing day and substrate. After 3 days of rearing, the bacterial communities were more similar to each other than the initial wastes (Figure 4A). Changes in the bacterial community were the largest within the first 6 days. The similarity between bacterial communities decreased again following 9 days. In both residues, most bacteria belonged to the genus *Lactobacillus.* Throughout the rearing period, the phyla *Proteobacteria* and *Bacteroidetes*, and the genera *Providencia*, *Dysgonomonas*, *Morganella*, and *Proteus* became more abundant than in the substrate.

#### Sterile Canteen Waste

Despite complete microbial inactivation by irradiation, the sterile canteen waste substrate had a similar bacterial community as the non-sterile canteen waste (Table 2 and Figures 3, 4B). Sterile canteen waste and canteen waste shared ZOTUs that accounted for 99.5% of the relative abundance (see Venn diagram in Supplementary Figure 6). Bacterial dynamics were also similar between sterile and non-sterile canteen waste (Figure 4B). The addition of BSFL to the sterile canteen waste led to a repopulation of the substrate to microbial counts similar to those determined in the non-sterile canteen waste (Table 2). *Lactobacillus* was also highly abundant in the sterile canteen waste residues, decreased in abundance during rearing, and *Providencia*, *Dysgonomonas*, *Morganella*, and *Proteus* became more abundant, but to a smaller extent compared to non-sterile canteen waste.

#### Substrates Without Larvae

**FIGURE 5:**
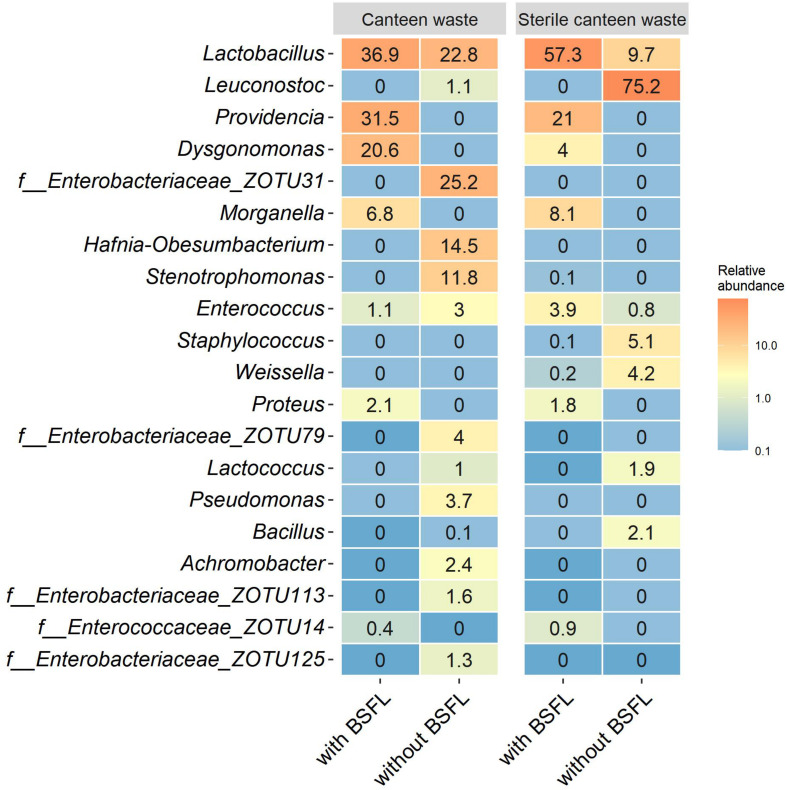
Bacterial community in canteen waste (sterile, non-sterile) with and without larvae following 12 days of rearing or storage. Heatmaps of the top 20 genera based on the relative abundance of ZOTUs in all samples. Relative abundances are the mean of replicate samples rounded to one digit. If no clear assignment to a genus was possible, the family assignment is shown together with the ZOTU.

The bacterial community of sterile canteen waste was similar to the initial bacterial community of the canteen waste substrates, after 12 days of storage (Figure 4B). However, for both sterile and non-sterile canteen waste, it differed noticeably from the bacterial communities in the rearing residues (Figure 5). The substrates stored without larvae had a higher bacterial richness (Table 2) and *Leuconostoc*, *Stenotrophomonas*, *Hafnia-Obesumbacterium, Lactococcus*, and *Enterobacteriaceae* were highly abundant but absent in the rearing residues (Figure 5). Genera that became more abundant in the rearing residues (see section “Substrates Without Larvae,” i.e., *Providencia*, *Dysgonomonas*, *Morganella*, and *Proteus)* were absent from the substrates stored without larvae.

### Correlation Among Bacterial Community, Rearing Performance, and Residue Composition

**FIGURE 6:**
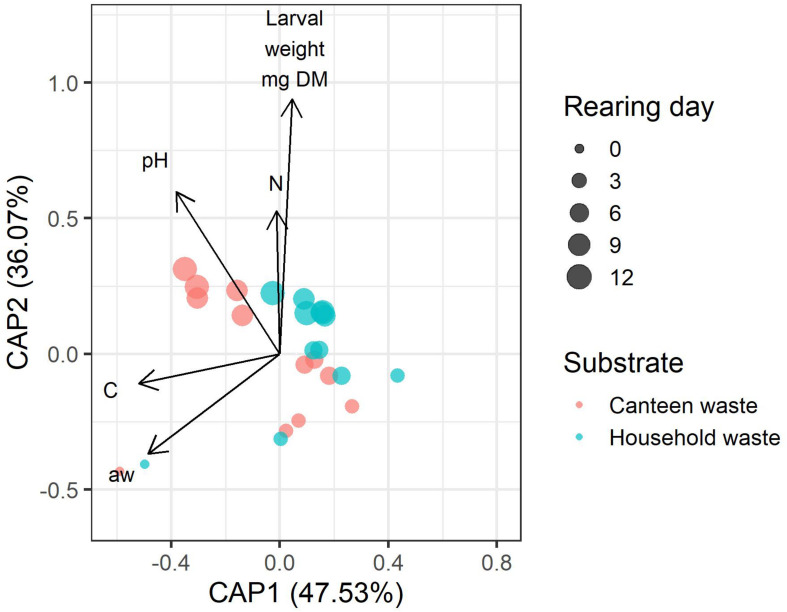
Distance-based redundancy analysis (dbRDA) biplot of canteen waste and household waste samples showing the correlation between the physicochemical properties and composition of the residue (pH, N, C, a_w_), larval weight, and the bacterial community. The length of the vectors indicates the relative importance of the parameter. The vector angle between variables indicates a correlation. Variables with smaller angles between vectors have a closer positive correlation. Perpendicular vectors indicate that there is no correlation. Vectors pointing in opposite directions indicate a negative correlation. Shorter distance between points indicates similarity between bacterial communities.

Bacterial community dynamics correlated with rearing performance and physicochemical properties and composition of the residue (Figure 6). Of all parameters, larval weight, and residue pH, carbon, nitrogen, and water activity had the lowest co-linearity and were used in distance-based redundancy analysis (dbRDA). The global dbRDA model and the first two axes were statistically significant and explained 83.5% of the variability in the bacterial community.

## Discussion

### Substrate Microbiota Contributes to Rearing Performance

We compared results for substrate and residue bacterial communities and BSFL rearing performance indicators between sterile and non-sterile canteen waste. We hypothesized that inactivation of the initial bacterial community in the rearing substrate will decrease rearing performance and reveal important members of the microbiota influencing improved rearing performance.

Our hypothesis was confirmed as results show that inactivation of microbes in the substrate reduced rearing performance (Figures 1, 2), suggesting that the initial substrate microbial community contributes to substrate decomposition and/or larval growth. The initial canteen waste microbial community was dominated by LAB (Figure 3), which is typical for fermented foods (Wu et al., 2018) and grain-based substrates (Wynants et al., 2019) with high contents of digestible carbohydrates (i.e., starch) (Table 1) and low pH (Table 2). LAB that have been shown to promote the growth of *Drosophila melanogaster* (Shin et al., 2016; Storelli et al., 2018), are routinely used as feed additives to promote the growth of farmed animals (e.g., poultry, pigs, and cattle) (Vieco-Saiz et al., 2019), and the addition of LAB (e.g., *Lactobacillus buchneri*) to the substrate has been shown to increase BSFL rearing performance (Somroo et al., 2019; Mazza et al., 2020). The mechanisms by which LAB promote growth are debated and still part of ongoing research, but suggestions include an increase of the metabolic capacity by fermentation of substrate carbohydrates into short chain fatty acids (as indicated by the low pH in the residue, Table 2), growth signaling, immunity, and protection and maintenance of stable gut microbiomes (Holzapfel et al., 2001). Interestingly, bacteria and fungi in the digestive tract or on the surface of larvae repopulated the high-energy electron beam treated canteen waste during rearing (Table 2) but rearing performance indicators remained nevertheless lower given the loss of the initial microbial community (Figures 1, 2).

Intact DNA after high-energy beam treatment interfered with further interpretation of bacterial dynamics in the sterile canteen residue. Despite lethal irradiation doses (Gold et al., 2020b), similar bacterial community between sterile and non-sterile canteen waste (Figure 3) after 12 days indicates that some bacterial DNA remained intact and was considered in the bacterial community based on DNA sequencing. As the bacterial communities identified in the sterile canteen waste residue may also include members without any major metabolic functions, these results should be interpreted with caution and are not further discussed. This finding agrees with recent research on the effect of irradiation on bacteria. Hieke and Pillai (2018) reported that *Escherichia coli* maintain their cell integrity post-irradiation, which protects DNA from denaturation. Since larvae and the associated microbiota digest bacteria (Gold et al., 2018), it is possible that the determined bacterial community becomes more representative of the viable bacterial community with increasing rearing duration.

### Rearing Performance Between the Two Food Wastes

While it has been recognized that both the rearing substrate nutrient composition and the microbial community (De Smet et al., 2018) composition influence rearing efficiency and reliability, previous studies typically emphasized only one aspect (Bruno et al., 2019; Klammsteiner et al., 2020) or considered both aspects but in isolation (Wynants et al., 2019; Gold et al., 2020a). We determined both substrate nutrient contents and bacterial communities and this over the rearing duration. We purposely used two rearing substrates with similar nutrient contents. Thereby we expected that the differences in rearing performance could be more easily attributed to the different substrate bacterial communities and could reveal members associated with rearing performance rather than showing the effect of nutritional differences.

Rearing performance was high with both food wastes (Figures 1, 2). We expected this based on the high nutrient contents (Table 1). Previous studies have reported lower bioconversion rates for food waste of 13.9–22.7% DM (Nyakeri et al., 2017; Lalander et al., 2019; Gold et al., 2020a) as compared to 23.5–28.0% DM in this study. Waste streams with low nutrient contents, such as digested waste water sludge, or cow and poultry manure, typically have even much lower bioconversion rates of 2.2–3.8% DM (Lalander et al., 2019; Gold et al., 2020a). Since the bioconversion rate is a key indicator determining the economics of insect rearing and the environmental sustainability of insect-derived products (Smetana et al., 2019), the results show that food waste could be an especially viable substrate for BSFL rearing.

Rearing performance was similar between the two rearing substrates with regard to conversion efficiency (Figure 1). However, larval biomass production (Figure 1) and waste reduction (Figure 2A) were higher for household than for canteen waste substrates. As demonstrated by the reduction in rearing performance due to the loss of the initial microbial community, differences in the initial substrate bacterial community could have contributed to the differences in this rearing performance. Notably, between the two substrates, there was a considerably higher waste reduction within the first 3 days of rearing (Figure 2A). However, as the two substrates shared most taxa, no conclusions can be drawn on bacteria that may explain the differences in rearing performance between substrates. It appears to be more likely that the disparity in rearing performance between substrates is due to different content and digestibility of nutrients. For example, non-fiber carbohydrates in household waste are likely more digestible for BSFL, considering that they were mostly comprised of glucose and fructose (Table 1). Pimentel et al. (2018) demonstrated that *Musca domestica* larvae can directly absorb glucose in the anterior and posterior midgut. Starch, however, requires catalysis before absorption and comprises all non-fiber carbohydrates in the canteen waste (Table 1) therefore being less directly digestible. In addition, household waste digestibility may have been increased by the onset of microbial substrate decomposition during storage (i.e., in the order of hours to days) at the household level (Albuquerque and Zurek, 2014). In contrast, canteen waste was collected on the same day of waste generation and stored at 4°C.

In summary, these findings demonstrate the challenge of unambiguously identifying the causes for differences in rearing performance despite comprehensive analysis of nutrient contents and bacterial communities. Differences in rearing performance could be due to differences in the low-abundance taxon or due to differences on the species level for the same genus. In addition, although Bruno et al. (2019) and Klammsteiner et al. (2020) concluded that bacterial gut communities are rather similar between substrates with broadly similar nutrient contents (Table 1), differences in bacterial community structure and function among substrates, as demonstrated by Zhan et al. (2020), may also have contributed to rearing performance differences.

### Common Fly Associated Bacteria Dominate the Rearing Residue

Microbial dynamics in the rearing residues of BSFL are still poorly understood. We studied bacterial community dynamics to identify taxa that were more abundant during BSFL rearing and that were absent in the controls (i.e., substrates stored under the same environmental conditions but without larvae). As suggested by previous researchers (Zhao et al., 2017; Ao et al., 2020), we hypothesized that bacteria enriched in the rearing residue contribute to substrate decomposition. This could imply that pure-culture bacteria and/or defined bacterial mixtures comprised of these bacteria from the residue could potentially increase large-scale BSFL rearing.

Previous studies have reported inconsistent results on the significance of altered substrate bacterial community during BSFL rearing (Bruno et al., 2019; Jiang et al., 2019; Cifuentes et al., 2020). Our study confirmed that BSFL rearing dramatically changes the substrate bacterial community and physicochemical properties and composition. For example, consistent with previous research, the pH increased in the residue during BSFL rearing (Table 2; Erickson et al., 2004; Lalander et al., 2014; Ma et al., 2018; Jiang et al., 2019; Wynants et al., 2019; Klammsteiner et al., 2020). Characteristic for food waste decomposition, the residue pH initially decreased due to the hydrolysis of proteins (Wu et al., 2018). Following 6 days of rearing, the pH increased beyond the initially value in the substrate, presumably due to the excretion of nitrogenous compounds by BSFL (e.g., uric acid) (Klammsteiner et al., 2020). Similar to the results of Jiang et al. (2019), ordination plots (Figure 4) revealed an obvious succession of the bacterial community throughout BSFL rearing. Consistent with previous research by Jiang et al. (2019) and Wynants et al. (2018), this study observed a reduction in bacterial richness (Table 2) in the residue in comparison to the substrate over the rearing duration. In contrast to Wynants et al. (2018) and Jiang et al. (2019), Bruno et al. (2019) did not observe significant differences in the bacterial community between substrates and residues, and Cifuentes et al. (2020) observed an increase in bacterial richness. Different abiotic (e.g., temperature, substrate nutrient content, and pH) and biotic (e.g., initial substrate bacterial communities) factors known to influence microbial ecology and the presence and stability of antimicrobial proteins by BSFL (De Smet et al., 2018; Vogel et al., 2018) may provide some explanation for discrepancies in findings among studies. Wynants et al. (2018) studied BSFL rearing with a variety of mostly grain-based substrates in several laboratory and industry-scale settings, Jiang et al. (2019) with food waste in an industry-scale setting, and Bruno et al. (2019) with a standard substrate for fly rearing, vegetables and fish waste in a laboratory setting (Table 3). Considering that substrate digestion by BSFL (Cai et al., 2018; Bruno et al., 2019) and other fly larvae (e.g., *Lucilia sericata*, Diptera: Calliphoridae) (Mumcuoglu et al., 2001; Lerch et al., 2003) decreases bacterial richness along the digestive tract (Gold et al., 2018; Vogel et al., 2018), different feeding rates, intervals, and larval densities among studies could be especially relevant (Table 3). One could expect that the substrate and residue bacterial community is altered to a greater extent by BSFL when less feed is provided per larvae (i.e., lower feeding rate and/or higher larval density). Our study and that of Jiang et al. (2019) had a 2–3-fold higher larval density and feed was provided less frequently when compared to Bruno et al. (2019), thus allowing more time for larval digestion (Table 3). Cifuentes et al. (2020) provided an insufficient rearing protocol to allow comparison with the other studies. A further study focusing more on microbial dynamics in BSFL under different rearing parameters is therefore recommended. However, considering the results of this and previous studies, and that large-scale rearing facilities may have higher larval densities [e.g., 4 larvae per cm^2^ by Dortmans et al. (2017)], a lower number of bacterial species can be expected in well-digested residues in comparison to the initial substrates.

**TABLE 3:** Literature summary of bacterial communities in BSFL rearing residues.

References	Substrate	Major families*	Major genera**	Feeding rate	Feeding interval	Larval density
				
				mg DM/day	–	BSFL/cm^2^
This study	Canteen waste Household waste	***Lactobacillaceae***, ***Enterobacteriaceae***, *Porphyromonadaceae*	***Lactobacillus***, ***Providencia*,** *Dysgonomonas*, ***Morganella***	22	One time	2.5

Jiang et al. (2019)	Food waste	*Corynebacteriaceae*, ***Bacillaceae***	*Corynebacterium*, ***Bacillus***, ***Lactobacillus***, *Enterococcus*	125	Every 2 days	2.9

Bruno et al. (2019)	Substrate for fly rearing	***Flavobacteriaceae***, *Comamonadaceae, Sphingobacteriaceae, Stenotrophomonas*	*Flavobacteriaceae, Comamonas*, ***Sphingobacterium***, *Stenotrophomonas*			
	Vegetable waste	***Lactobacillaceae***, *Methylocystaceae*	***Lactobacillus***, *Methylocystaceae*			
				*ad libitum*	Every 1–2 days	1.0
	Fish waste	***Planococcaceae***, *Clostridiales, Bacilli*, ***Peptostreptococcaceae***, *Clostridiaceae*	*Planococcaceae, Clostridiales, Bacilli, Tissierella*, ***Clostridium***			

Wynants et al. (2018)	Fruit and vegetable waste	***Enterobacteriaceae***, ***Bacillaceae***, *Planococcaceae*	*Cosenzaea*, ***Bacillus***, ***Morganella***, ***Providencia***, ***Sporosarcina***			
				*ad libitum*	Every 3–4 days	–
	Supermarket/ restaurant waste	*Lactobacillaceae, Enterobacteriaceae*	***Lactobacillus***, ***Morganella***, ***Providencia***			
				
	Poultry blood	***Lactobacillaceae***, ***Enterobacteriaceae***, *Clostridiaceae*, ***Peptostreptococcaceae***	***Lactobacillus***, *Buttiauxella*, ***Clostridium***, *Pediococcus, Peptostreptococcus*			
	Poultry manure	***Bacillaceae***, *Carnobacteriaceae*	***Gracilibacillus***, *Virgibacillus, Lentibacillus*, ***Atopostipes***, ***Amphibacillus***, ***Bacillus***, ***Oceanobacillus***			

	Brewery waste, fruit waste	*Pseudomonadaceae, Sphingobacteriaceae*, ***Flavobacteriaceae***, ***Bacillaceae***	*Pseudomonas*, ***Sphingobacterium***, ***Flavobacterium***, *Myroides*, ***Bacillus***, ***Oceanobacillus***, *Mucilaginibacter*			
				
	Vegetable waste, mill by-products, yeast	***Bacillaceae***, ***Planococcaceae***	***Gracilibacillus***, ***Oceanobacillus***, ***Sporosarcina***			
				
	Fruit and vegetable waste, brewery waste, former food products, food waste	***Bacillaceae***, *Pseudomonadaceae, Carnobacteriaceae*, ***Flavobacteriaceae***, ***Bacillaceae***	***Bacillus***, ***Amphibacillus***, *Thiopseudomonas*, ***Atopostipes***, ***Flavobacterium***, *Gracilibacillus*			

Shelomi et al. (2020)	Soy pulp, Canteen waste	*Cellvibrionaceae, Cytophagaceae, Caulobacteraceae*	*Cellvibrio, Leadbetterella, Brevundimonas*	*ad libitum*	Daily	–

Families and genera identified in at least two studies are highlighted in bold. The bacterial community was identified in all studies by 16S rRNA gene sequencing. * relative abundance >10%, in order of descending relative abundance. ** relative abundance >5%, in order of descending relative abundance; whenever the genera could not be identified, the lowest available taxonomic rank (i.e., family or class) is reported.

Genera in the residues at the time of larval harvest are ubiquitous in the environment (e.g., soil, water, digestive tracts of humans, and farmed animals) and have been previously identified in different life stages of the BSF (Table 3; Bruno et al., 2019; Cifuentes et al., 2020; Klammsteiner et al., 2020; Raimondi et al., 2020; Zhan et al., 2020), *Musca domestica* (Zurek et al., 2000; Su et al., 2010; Gupta et al., 2012), and *Lucilia sericata* (Singh et al., 2014). Bacteria commonly associated with BSFL residues (Table 3) are from the *Lactobacillaceae*, *Bacillaceae*, *Enterobacteriaceae*, *Planococcaceae*, and *Flavobacteriaceae* families. One possible explanation for the recurrence of these taxa in fly larvae residues is through the transfer of intestinal commensal bacteria with larval secretions and excretions into the residue (Zhao et al., 2017; Storelli et al., 2018). The results of this study support this hypothesis. Recurring taxa in the residues belong to the phyla *Firmicutes*, *Proteobacteria*, and *Bacteroidetes*, which are the main intestinal bacteria in fly larvae (Zurek et al., 2000; Jeon et al., 2011; Boccazzi et al., 2017; Scully et al., 2017; Cifuentes et al., 2020; Liu et al., 2020). *Providencia* spp., *Dysgonomonas* spp., *Morganella* spp., and *Proteus* spp. present in the BSFL residues, and absent in controls without larvae, are highly abundant in the BSFL guts (Ao et al., 2020; Cifuentes et al., 2020; Klammsteiner et al., 2020; Raimondi et al., 2020). Identification of *Dysgonomonas*, *Providencia*, *Morganella*, and *Proteus* in the posterior midgut of BSFL suggest that members of these genera may survive gut passage (Bruno et al., 2019). The one-time feeding regime, low feeding rate, and high larval density in our experiments could have contributed to the more pronounced appearance of these genera than in previous studies (Bruno et al., 2019; Wynants et al., 2019), as the residue presumably passed the digestive tract more often than that at higher feeding rates and frequency and lower larval densities. However, it is important to bear in mind that these genera are typically present at low abundance (<0.01% in this study) in BSFL substrates (see Figure 3; Bruno et al., 2019; Shelomi et al., 2020). Consequently, the proliferation of these genera during BSFL rearing could also have been in part due to the observed changes in the residue physicochemical properties and composition, and not only the secretions/excretions of BSFL. For example, members of the genera *Proteus*, *Providencia*, and *Morganella* are involved in urea hydrolysis and thus may benefit from the nitrogenous compounds excreted by BSFL (Manos and Belas, 2006; Klammsteiner et al., 2020).

*Dysgonomonas*, *Providencia*, *Proteus*, and *Morganella* have important functions in the life cycle of fly species. Despite their prominence in the digestive tracts and residues, the ways in which they influence larvae and substrate decomposition are still poorly understood. Studies focusing on the role of these bacteria in BSFL rearing do not exist. *Morganella* spp. and *Providencia* spp. are typically transferred between generations in several fly species (Su et al., 2010), and *Proteus spp.* have been isolated from the egg surface of the BSF (Mazza et al., 2020) and the digestive tract of *Musca domestica* (Su et al., 2010; Gupta et al., 2012). One function of these bacteria appears to be stimulation of the fly ovipositor by the release of volatile compounds. Different species of these genera, such as *Proteus mirabilis*, have been shown to control fly oviposition in *Lucilia sericata* (Ma et al., 2012; Tomberlin et al., 2012; Uriel et al., 2020) and *Cochliomyia hominivorax* (Diptera: Calliphoridae) (DeVaney et al., 1973; Eddy et al., 1975). In addition, *P. mirabilis* may repel bacteria that are detrimental to larval development. *P. mirabilis* is associated with *Lucilia sericata* and exerts bactericidal effects (Erdmann and Khalil, 1986), but the antimicrobial excretion/secretion from *Lucilia sericata* is not active against *P. mirabilis*, suggesting a symbiotic host-microbe relationship (Barnes et al., 2010). In addition, bacteria may support the decomposition of substrate constituents (Zhao et al., 2017). Bruno et al. (2019) identified *Dysgonomonas* and Ao et al. (2020) *Providencia* as major genera in the BSFL digestive tracts and proposed that their members could be involved in the digestion of hemicellulose and proteins and lipids, respectively. Similarly, *Dysgonomonas* and Providencia could contribute to nutrient decomposition in the residue. However, it should be noted that there is considerable uncertainty with these claims as they are based on findings for phylogenetically different well-studied insects (e.g., honeybees and termites), correlations between bacterial communities and environmental parameters (e.g., substrate nutrients), or functional predictions based on DNA sequencing, which may not provide direct evidence for bacterial community functional capacities.

We demonstrated that there is considerable overlap between bacterial communities in BSFL residues and digestive tracts. We hypothesize that some members of these genera may influence substrate decomposition and larval development, and therefore have the potential to increase the performance of large-scale BSFL rearing. A natural progression of this work is to isolate members of these genera from residues or larval digestive tracts, and assess their potential to increase rearing performance by adding them to the rearing substrates *in vivo* (Yu et al., 2011; Xiao et al., 2018; Rehman et al., 2019; Somroo et al., 2019; Mazza et al., 2020) and *in vitro* (Gold et al., 2020c). Further research is needed to better understand the variable effectiveness among bacterial species (Mazza et al., 2020), and among strains of the same species (Yu et al., 2011), in influencing rearing performance under variable biotic and abiotic conditions typical in practice. These studies should use or imitate large-scale rearing conditions (e.g., larval densities) to ensure maximum transfer of results into practice. Even though we used realistic rearing conditions (i.e., feeding rates and larval densities), residue temperatures (27–30°C) influencing bacterial communities in BSFL (Raimondi et al., 2020) were below those found in large-scale rearing (e.g., 33–45°C) (Bloukounon-Goubalan et al., 2019) due to the bench-scale nature of our study.

### Implications for Product Safety

Despite not being the main focus of this research, our results present relevant findings regarding BSFL rearing product safety. BSFL substrates may have pathogenic microbes (Erickson et al., 2004; Lalander et al., 2013) and since BSFL live within their rearing substrate and pass it through their digestive tract, pathogenic microbes inside or on the harvested larvae are a hazard for product safety. Such pathogenic microbes in the harvested larval biomass can be eliminated by thermal or non-thermal inactivation technologies. An alternative approach could be the inactivation of pathogenic microbes in the substrate before BSFL rearing, for instance by irradiation. Our results suggest that such an approach may greatly decrease rearing performance. Future research should be undertaken to mimic more realistically rearing facility substrate inactivation technologies (e.g., pasteurization) and conditions (e.g., time, temperature). Some technologies may only lead to partial microbial inactivation and at the same time reduce particle size and increase nutrient digestibility, impacting positively on rearing performance. However, considering that fly larvae may live in close association with pathogens such as *Providencia rettgeri*, *P. mirabilis*, and *Morganella morganii*, post-harvest treatment of the residue (e.g., composting) and larval biomass (e.g., heat treatment such as pasteurization) may still be the most efficient and reliable approach.

## Conclusion

Sustainable mass rearing of BSFL for feed and food applications requires efficient and reliable process performance. Complementing previous work on the larval microbiota, this study set out to identify bacterial taxa in two food waste rearing substrates and residues that are potentially associated with rearing performance. As expected, considering their high nutrient content, rearing performance was high with canteen and household food waste substrates, underlining their potential for efficient insect production. A loss of the initial food waste microbiota, dominated by lactic acid bacteria, decreased rearing performance, indicating that initial substrate microbiota influence the complex bioconversion process. Furthermore, the rearing performance could also be influenced by bacteria in the rearing residue. Rearing duration decreased the bacterial richness and changed the physico-chemical properties and composition of the residue, and typical members of the larval intestinal microbiota (that is, *Providencia, Dysgonomonas, Morganella*) became more abundant, suggesting their transfer into the residue through excretions. The present study provides a scientific basis for future studies that should isolate these bacteria and assess their true role in influencing rearing performance.

## Data Availability Statement

All original data presented in the study is publicly available. This sequencing data can be found at: https://www.ncbi.nlm.nih.gov/PRJNA646490. All other data and analyses can be found at: https://github.com/MoritzGold/BSFL_residue_microbiota.

## Author Contributions

MG: conceptualization, methodology, investigation, formal analysis, visualization, writing – original draft, and funding acquisition. FA: conceptualization, methodology, investigation, formal analysis, visualization, and writing – review and editing. CZ: conceptualization, supervision, project administration, funding acquisition, and review and editing. JZ: writing – review and editing. AM: conceptualization, supervision, project administration, writing – review and editing, and funding acquisition. All authors contributed to the article and approved the submitted version.

## Conflict of Interest

The authors declare that the research was conducted in the absence of any commercial or financial relationships that could be construed as a potential conflict of interest.
