# Supplementary material for: Identification of Bacteria in Two Food Waste Black Soldier Fly Larvae Rearing Residues
Source: Front Microbiol. 2020 Nov 23;11:582867. doi: 10.3389/fmicb.2020.582867 (PMC7719680; doi:10.3389/fmicb.2020.582867)
Supplement: Supplementary file 1 [file Data_Sheet_1.pdf]

**Identification of Bacteria in Two Food Waste Black Soldier Fly Larvae Rearing Residues**

**Supplementary Material**

**Supplementary Table 1:** Pictures of the fresh and homogenized food waste substrates used for BSFL rearing.

| Sub-strate | Fresh | Homogenized |
|---|---|---|
| Can-teen and sterile can-teen waste | 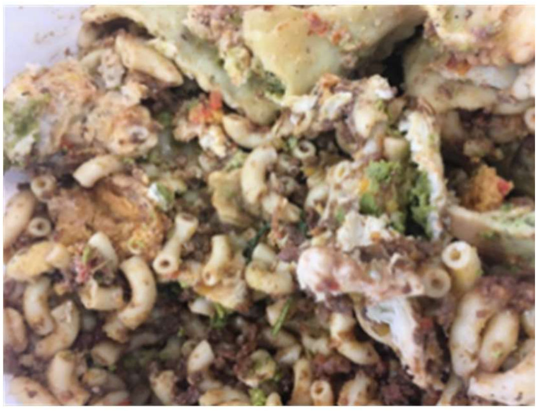 | 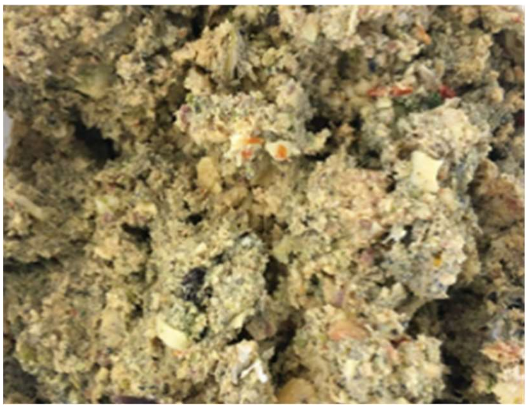 |
| House-hold waste | 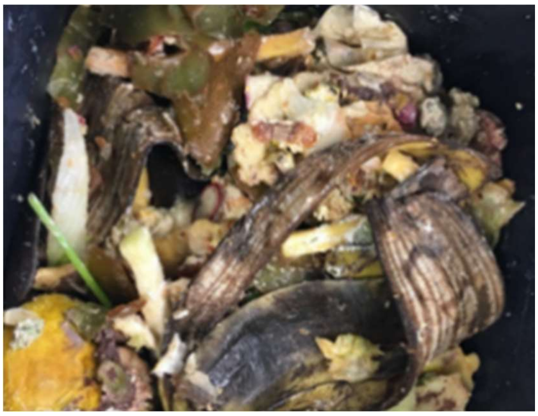 | 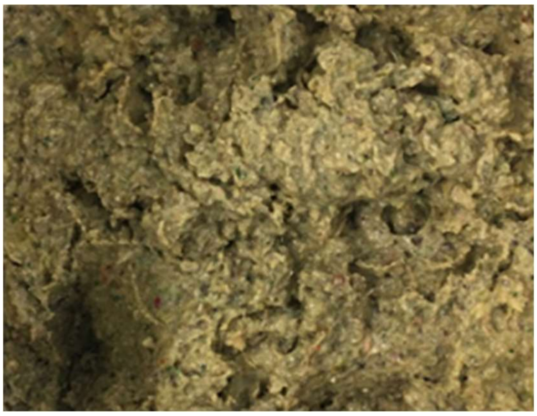 |

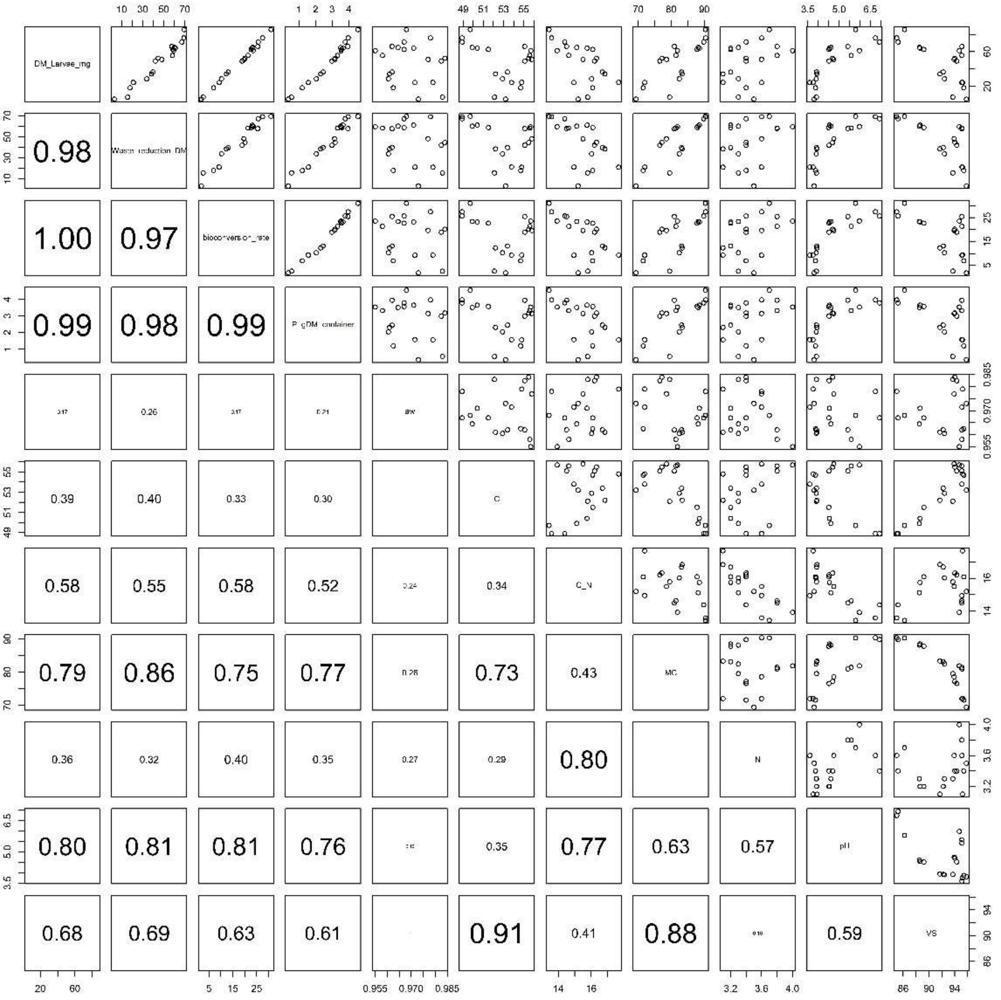


**Supplementary Figure 1:** Pearson correlation matrix to identify co-linearity between rearing performance metrics and physio-chemical residue composition parameters before Distance-based redundancy analysis (dbRDA). Correlation coefficients −0.7 < r <0.7 indicate strong co-linearity.

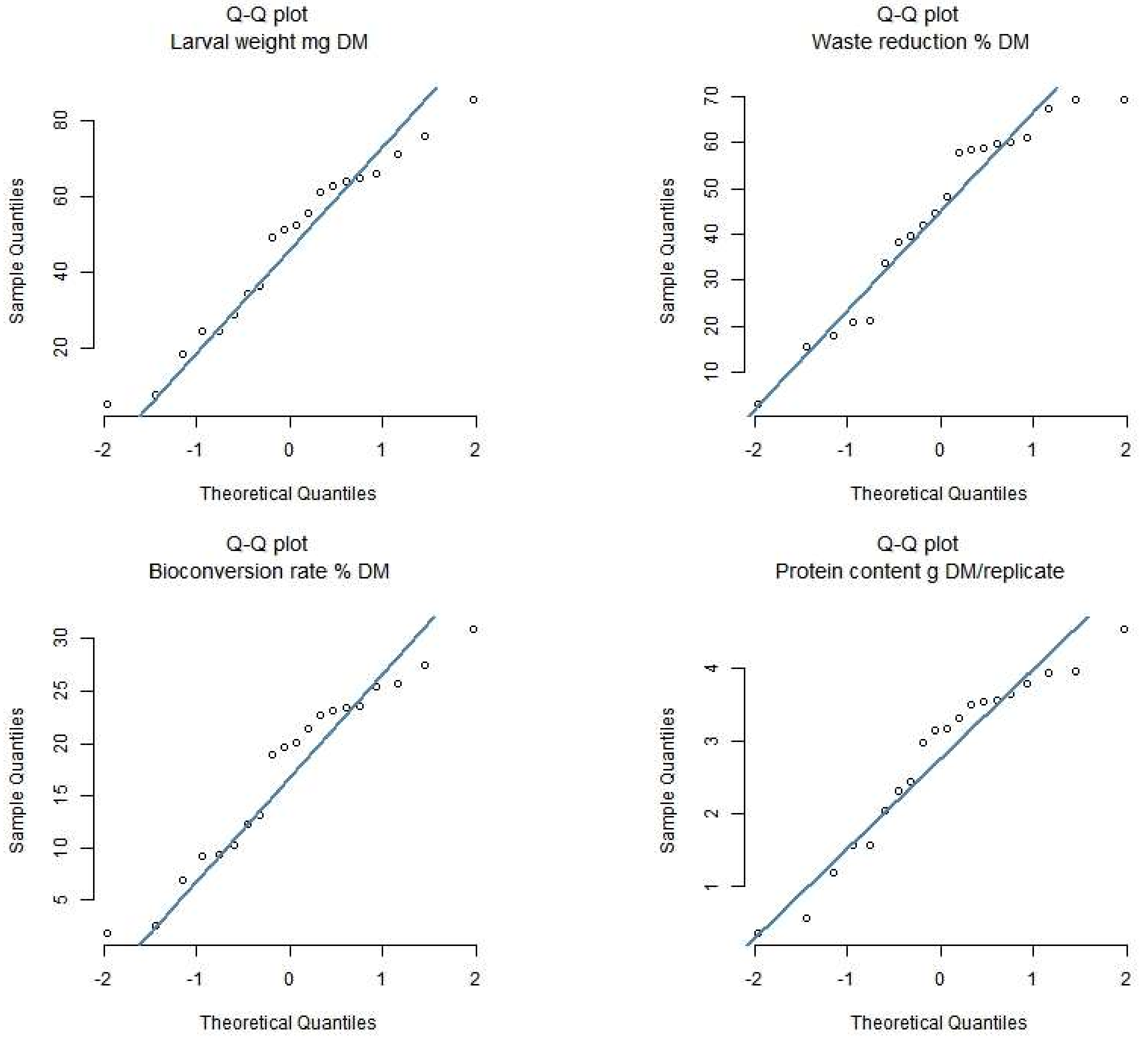


**Supplementary Figure 2:** Q-Q plots of performance indicators to assess normality. Larval weight (top, left), waste reduction (top, right), bioconversion rate (bottom, left) and protein content (bottom, right).

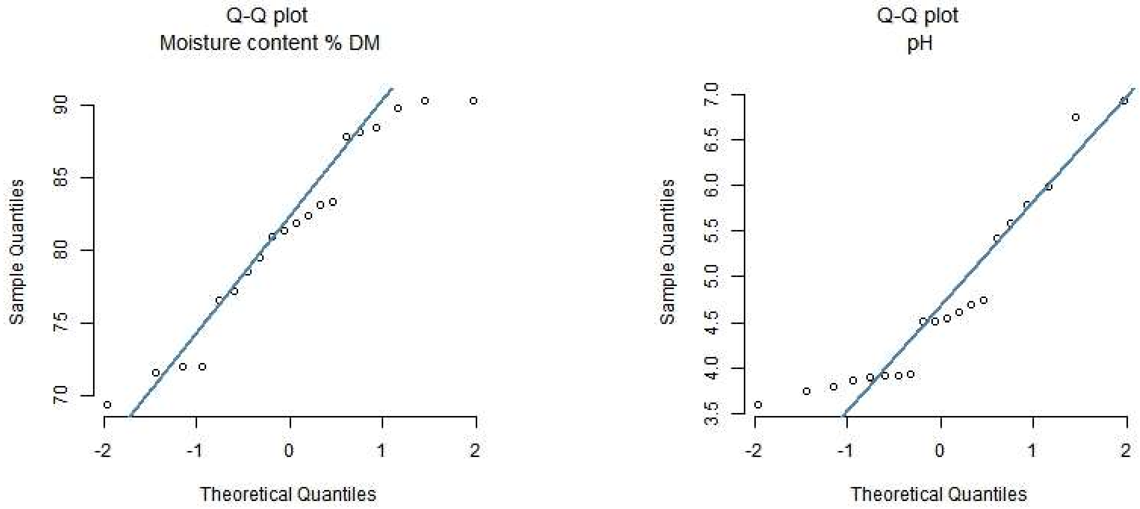


**Supplementary Figure 3:** Q-Q plots of physio-chemical residue composition parameters to assess normality. Moisture content (left) and pH (right) had a strong correlation with rearing performance indicators (see Pearson Pearson correlation matrix)

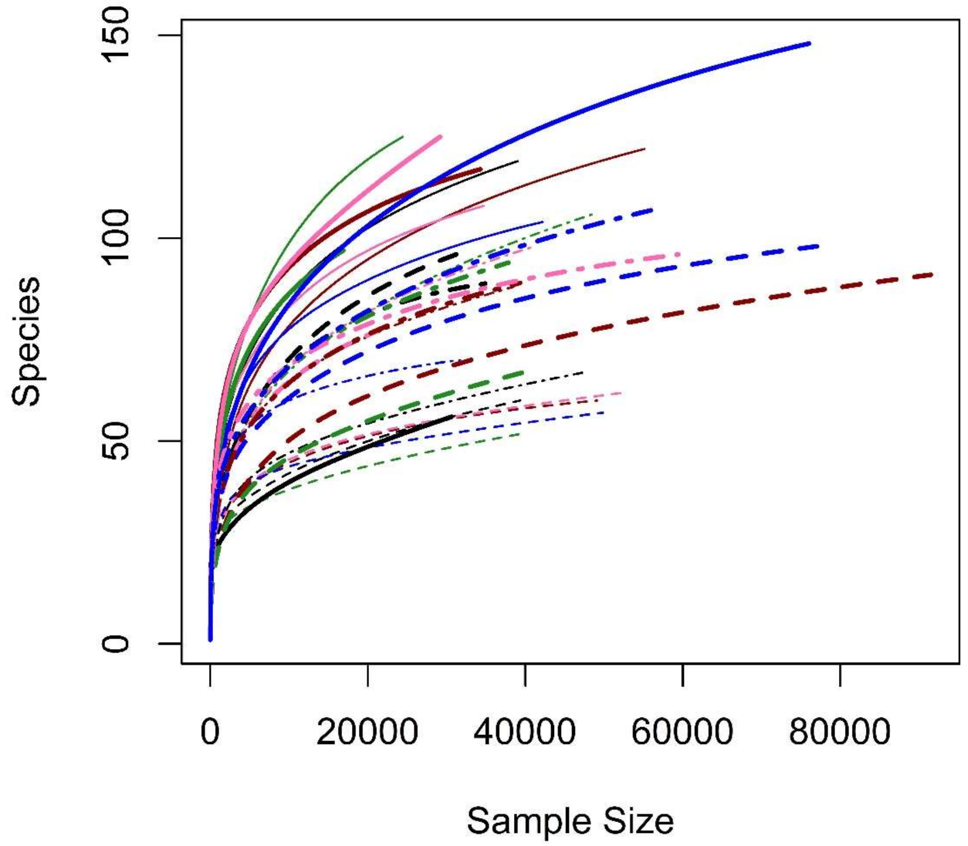


**Supplementary Figure 4:** Rarefaction curves of all substrate and residue samples. Colors and line types represent the different samples.

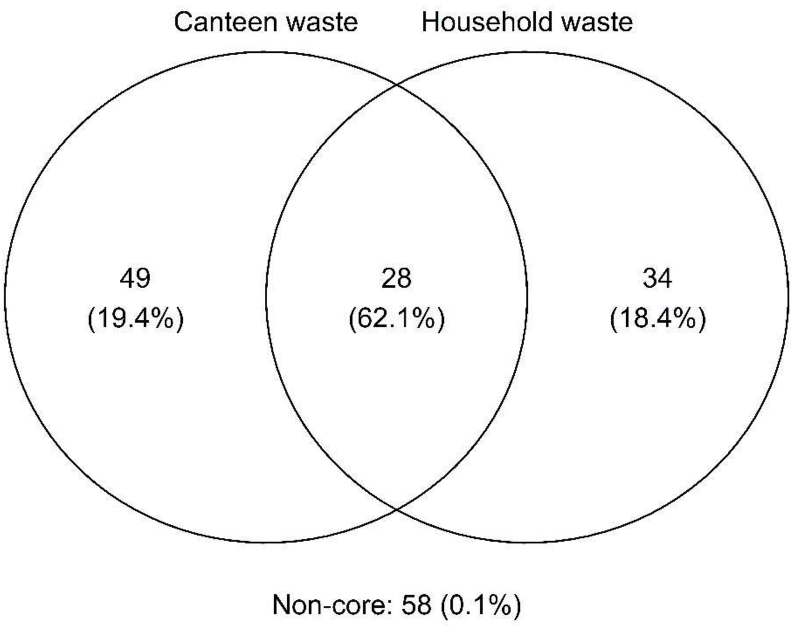


**Supplementary Figure 5:** Venn diagram showing shared ZOTUs between the canteen and household waste rearing substrates. Venn diagram was determine with the ampvis2 package (frequency cut-off >80% and abundance cut-off > 0.01%).

**Supplementary Table 2:** Carbon, water activity and temperature in the substrates and residues.

| Substrate | Day | Carbon | Water activity | Temperature |
|---|---|---|---|---|
| | | % DM | - | °C |
| ***With BSFL*** | | | | |
| **Canteen waste** | 0 | 55.9† | 0.98† | n.a. |
| | 3 | 53.4 (0.2) | 0.97 (0.0) | 28.1 (0.3) |
| | 6 | 54.4 (0.6) | 0.97 (0.0) | 29.2 (0.4) |
| | 9 | 55.5 (0.3) | 0.98 (0.0) | 29.8 (0.4) |
| | 12 | 55.5 (0.3) | 0.96 (0.0) | 29.8 (0.4) |
| **Sterile canteen waste** | 0 | 55.9† | 0.98† | n.a. |
| | 3 | 53.7 (0.9) | 0.98 (0.0) | 28.4 (0.1) |
| | 6 | 54.0 (0.2) | 0.96 (0.0) | 28.9 (0.4) |
| | 9 | 54.1† | 0.96† | 29.1 (0.1) |
| | 12 | 54.5‡ (0.3) | 0.96† | 29.1 (0.1) |
| **Household waste** | 0 | 51.7† | 0.99† | n.a. |
| | 3 | 52.4‡ (0.3) | 0.98‡ (0.0) | 28.2 (0.2) |
| | 6 | 52.8 (0.6) | 0.96 (0.0) | 29.4 (0.5) |
| | 9 | 50.6 (0.8) | 0.97 (0.0) | 29.5 (0.2) |
| | 12 | 49.2 (0.5) | 0.97 (0.0) | 29.9 (0.1) |
| ***Without BSFL*** | | | | |
| **Canteen waste** | 12 | 56.0‡ (0.3) | 0.97 (0.0) | 28.8 (0.0) |
| **Sterile canteen waste** | 12 | 53.1 (0.4) | 0.96 (0.0) | n.a. |

In parenthesis: standard deviation for samples where n ≥ 3, differences between analyses where n = 2

† n=1, ‡ n=2, n.a. = not analysed
